# Indents

**Published:** 1911-01-15

**Authors:** 


### INDENTS.

**☞Brief Articles of Technical or Scientific Value will receive notice and comment. Contributions invited.**

*A Method of Obtaining Primary Adhesion For Plates.*

One of the most annoying things to dentists is to make a plate for a patient and find that it will not *stay up*.

Often the roof of the mouth is flat, and not at all favorable for primary adhesion; in spite of all the precautions we take, such as relieving hard spots and compressing soft spots, the patient comes back a few days after the insertion of the denture and tells us that the plate will not stay in place; that no sooner does he open his mouth than the plate drops out. This result is specially frequent with aluminum plates.

The writer has remedied a number of such cases by vulcanizing soft, velum rubber in the vacuum chamber in the following manner: First, a deep undercut is made in the walls of the vacuum chamber: second, the chamber is filled with soft bees wax, and an impression is taken of the space occupied by the vacuum chamber upon the roof of the mouth. The plate is removed and the wax trimmed as many times as is necessary. The wax should not extend beyond the margin of the vacuum chamber when the case is ready for flasking. The flasking is done in such a way that, when the two halves are separated, the palatal surface of the plate will be exposed. Every dentist knows how to flask, and details here are not necessary.

After separating the flask, the wax is removed by means of boiling water, and packing is the next step. In packing, ordinary vulcanite is packed in the undercut, so as to insure firm attachment to the plate, and the rest of the chamber is filled with soft velum rubber. Here, too, it is advisable to open the flask, after it has been pressed and remove the excess rubber, if any. The flask is closed, bolted and vulcanized. After vulcanization the plate only needs pumicing with a brush and the final polish.—Dr. Golden, in *Dental Brief*, October 1910.

*Gingivitis in Diabetes.* The importance of mouth symptoms in the acute infections, such as scarlet fever, diptheria, and measles, is recognized. It is less generally known, however, that in many constitutional conditions the mouth secretions and the mucous membranes covering the gums, cheeks, tongue, etc., furnish early and positive data for diagnosis. It has been proved that gingivitis is an early and prominent symptom in many cases of diabetes mellitus, and continues throughout the course of this disease. Many good books on general medicine ignore this fact, however. Osler, who seldom misses a point, makes no mention of it in diabetes, but does say that "The tongue is usually dry, red and glazed, and the saliva scanty; the gums may become swollen, and in the later stages of the disease aphthous stomatitis is common." J. Redier makes the definite statement that in diabetes the saliva contains sugar, which undergoes lactic fermentation, in consequence of which both the teeth and the gums suffer. G. Lemoine thinks that the complications of diabetes involving the digestive apparatus are far from rare, especially the mouth complications. "Among the more frequent," he says, "is gingivitis of the simple conjestive suppurative, or expulsive type; this gingivitis seems due to the rapid multiplication of microbes in the buccal cavity." Ludwig Brandt says that alveolar atrophy begins around the incisors of the lower jaw, which have the weakest roots and the most delicate alveolar lamelle. He mentions especially the influence of disorders of nutrition on pyorrhea, and cites as constitutional causes mercury taken as a medicine or introduced into the system by constant handling, diabetes, rheumatic and acute arthritides scurvy, syphilis, tuberculosis and certain nervous diseases, such as neurasthenia, tabes, and progressive paralysis.

The first symptom of gingivitis is usually a sensation of itching and burning in the gums, which is sometimes disquieting enough to bring the patient to the physician, when careful examination may discover one or more loose teeth. Suppuration may not be present at this time. At a later stage little or no pain is present, but there is marked retraction of the gums, loosening of all the teeth, and the oozing of pus from the tooth sockets. In later stages the teeth drop out or must be extracted.

It is interesting to note that the onset of both pyorrhea and diabetes takes place most frequently between twenty and thirty years of age, and that males are the more frequently effected. The upper jaw is more resistant than the lower, and the disease usually begins around the lower incisors, as already mentioned. The treatment is of course, essentially constitutional, supplemented by local applications.

It becomes a matter of extreme importance, therefore, that the general practitioner shall examine the mouths of all patients, taking careful note of the mucous membrane of the cheeks, beneath the tongue, on the tongue itself, the roof of the mouth, and especially the gums.—*A. M. A. Journal,* May 7, 1910.

*Pyorrhea with Systemic Complications.* On Friday I had a telephone call from a young woman who said that she had been trying to have her mouth treated for pyorrhea, and she had been sent from pillar to post, and had been finally recommended to see me. I gave her an appointment for Saturday. When she arrived I was very busy—so busy that I felt I would have to ask her to call again. I went into the waiting room and found a young woman of twenty-six whom I did not want to dismiss summarily. I asked her to let me look at her mouth casually, over by the window. I found it to be a case of acute pyorrhea alveolaris, pus flowing freely from every socket in the mouth—as marked a case as I have ever seen. I quickly recognized the pre-disposing cause of that pyorrhea to be heart disease. There was an impairment of the heart to such an extent that the entire mucous membrane of the mouth was cyanosed—and anyone who has examined heart disease recognizes that disease at once. It was interesting to me to demonstrate to a physician, whose wife I was treating at that moment, the value of mouth examination in diagnosis; so I asked the young woman to be seated, returned to my office, and recited the facts to this doctor, asking him if he were willing to examine the patient's heart. He made an examination with his stethoscope and at once corroborated the diagnosis of her condition.

He sent me a letter going over the facts in the case, because I had him make an appointment with the young woman to examine her carefully. Upon this examination of her heart he told me that he did not think it to be an organic lesion. Neither did I. A careful examination of the patient, who is a bookkeeper, showed that the heart disease had been caused by the most common constitutional exciting cause that I believe we find in large cities to-day, *i. e.*, intestinal auto-intoxication. The woman gave a history of having a movement of the intestines sometimes not more than once in six or seven days; and I have no doubt that this auto-intoxication has affected the muscles of the heart, as was borne out by the examination. The history of the patient shows the development of the pyorrhea as a result not directly of the auto-intoxication, but of the impaired nutrition due to the fact of the heart being unable to perform its functions properly. I am reciting this case solely for one purpose: I would have liked to read in detail the result of the examination of this patient, because it is a typical one of a rare type. My object, however, is to bring out the fact that if we are to be intelligent practitioners, it is ridiculous for us to say, "Here is a case of pyorrhea," and nothing more. Each case of pathologic disturbance of the mouth should be diagnosed and treated on its own merits, and the diagnosis should be made conclusive before intelligent treatment can be pursued. This is the important step that we must learn as dentists, if we want to receive the respect of any intelligent physician.—Dr. M. L. Rhein, in *Cosmos*, page 1137, October 1910.

*How to Gargle.* To the needy ones this is a suggestion how to be able to gargle. It especially refers to the large number of people who say: "I can't gargle—it chokes me." One can gargle as deep down the throat as needful by pressing the palm of the left hand upon the epiglottis, and quite hard, directly above and against the thyroid cartilage. Do not press upon the cartilage sideways, in a manner of choking, but *against* its *anterior* part only, covering about two inches of its *outer* surface. Instead of the palm of the hand, one may use the tips of the thumb and of all the fingers of the left hand closely arranged in a curve and adapting them to the trachea. My suggestion has not failed in the most difficult cases, and should be tried until it is correctly followed. Patients should receive these instructions and *be shown how* to gargle, as above described. —M. J. Emelin, D.D.S., *The Dental Brief.*

*Value of Good Teeth.* We cannot have thorough mastication without good teeth; without thorough mastication we cannot have perfect digestion; without perfect digestion we cannot have perfect assimilation; without which we cannot have nutrition, which means health. Without good health what becomes of our disposition? It becomes degraded, harsh and sour; in other words, we become "undesirable citizens."

Investigations show that more bright children have better teeth than dull children, and that the average age of children with good teeth in any grade is *less* than that of children with poor teeth in the same grade. In Germany it has been demonstrated that when children's teeth were put in good condition they became physically stronger, secured a higher average in their studies, were easier to control and were apparently happier.—Dr. J. C. Watkins in *Brief.*

*The Seat of Hunger and Thirst.* Whether the feeling of hunger has its seat in the stomach and thirst in the throat is a question that most people take for granted, but it has been the subject of much scientific controversy. According to Dr. Valenti, an Italian physician, the seat of both hunger and thirst is in the gullet. In the *Archivi di Biologia* he publishes an article dealing with an experiment which he believes proves his contention. He found that a cocaine injection in the upper part of the alimentary canal resulted in instant suppression of the feeling of both hunger and thirst. He tried it on a dog, which refused to eat or drink for five days, after which the drug had worked itself out. Savages, however, knew long before Dr. Valenti that the chewing of cocoa leaves renders the gullet insensible and kills any desire for food or drink.—*Dental Brief.*

*Massaging.* Massage has its advantages as a test to determine whether or not any particles of the deposits are left, but it has a disadvantage in the earlier stages in that it presses out this lymph, forces it from the pocket and prevents the formation of ciccatricious tissue which would otherwise have taken place. I agree with the Doctor that this is the most important condition with which we have to deal in the mouth today. Oral prophylaxis is the subject before us. It is the subject with which we have to deal.—Dr. Worthly in *Western Dental Journal.*

*What the College Does.* The college does not make a student a skilled physician or a successful dentist. It simply gives to its graduates the tools by which, if properly used, they may become eminent and useful in their calling. What he becomes after his graduation the student makes himself, whether it be an ornament to his profession or a disgrace to the community. The kit may not be a full kit, and some tools may prove defective. It is a fair outfit, however, and if the student has in himself the real stuff that makes for success, he can readily make up the deficiencies as they develop.—T. Trueman in *Dental Brief.*

*Tin Models, Hollow, Cast From Impression.* Dr. J. J. Stetzer, of Philadelphia, has devised an ingenious way of producing hollow tin models direct from the impression, for use in vulcanite work. It is claimed that rubber vulcanized in contact with tin has a harder and more dense surface than when vulcanized in contact with plaster. Dr. Stetzer claims that the tin model, being harder and more resistant to pressure, ensures a better fit. A solid model is not readily removed from the vulcanized denture if there are any undercuts. Dr. Stetzer paints inside the impression with melted paraffin, endeavoring to give a thin, even coat. Then, after adjusting sprues as would be done in casting a denture, invests in a flask fitting a pressure casting machine, and proceeds to make a tin cast, just as he would if making a cast denture. When the cast is made and the investment cold, he carefully removes the investment from the inside of the casting, being careful not to disturb that which formed the impression, and replaces it with plaster. This procedure is essential, as the casting is very thin and easily injured. When this is hard the impression is removed, leaving a model with a cast tin face scarcely thicker than heavy tin foil, and readily removed from the vulcanized denture. The pressure used in casting the tin forces it into every line and mark of the impression, producing a more accurate model than is usually obtained with plaster. The impression is taken with investment material.—*Dental Brief.*

*Vulcanized Rubber, Its Chemical Formula.* Recent experiments indicate that vulcanized rubber is a definite chemical compouud with the formula C10H16, S2Cl2). This was determined by treating Para rubber with sulphuric chlorid. *Franklin Institute Journal*, October, 1910.

*Embalming the Apical Ends of Pulps.* Place a drop of glycerol on a glass slab. The glycerol contains one-half of one per cent. of formaldehyde. Add to it a piece of thymol the size of a grain of wheat and an equal amount of alum. Mix. By adding the thymol in this manner it is kept from flying when it is crushed. At this stage the mixture will be too thin for use and oxide of zinc should be added until the mixture is as thick as a stiff cement.

The ingredients of this mixture, as everybody knows, have been used by many for a long time. The point of interest is that by mixing it fresh each time it is used, it is much more efficacious. When mixed immediattly before using, no difficulty will be found in embalming the apical ends of pulps, even in tortuous canals.

By keeping at hand the four small bottles containing the elements of this mixture, little space is occupied and little more trouble involved than when the mixture is kept, already prepared, in a larger bottle. The freshly mixed preparation is efficacious where results with the other would be doubtful.—Dr. W. X. Heckard in *Digest.*

*Our Poison Squad.* The world's poison squad consists, in a large degree, of weak, puny children, made yet weaker by poor food, and further debilitated by the vitiated secretions of their own mouths. For their poisons are not administered at intervals in studied doses, but with every mouthful of food, with every involuntary swallow, the system is inoculated with virus, which, reaching a vantage point, may cause the death of the poor little mortal. Many a little grave would be tenantless and many a sad home would still echo to childish laughter had the crusade for Oral Hygiene been preached earlier. To the uninitiated this may sound somewhat sensational, but the truth of the matter is that it can be demonstrated that an unclean mouth is actually responsible for deafness, defective eye sight, dullness, anemia, catarrhal disorders leading to dispepsia and to consumption, and, indirectly, juvenile criminality, all these the result of autointoxication; and the child with an unclean mouth is a dangerous menace to his neighbors by reason of the direct dissemination of disease germs, or by the pollution of the atmosphere.—Bessie B. Bennett in *Brief*.

*Brushing the Teeth.* The first thing on rising, reach for the tooth brush, and brush the teeth before eating, because the act of eating and the excursions of food over the teeth push these particles under the free margin of the gum, and you will never get them out if you don't get them out before you eat. I am sure of that and I did not become sure until I had been doing it for forty years, or nearly that, and I have been advising it for thirty years. I don't think that I can be said to have jumped at that conclusion.

The next thing after eating a meal is to rinse the mouth and rinse it vigorously. The next thing is to pick the teeth. And now, Dr. James, in the female seminaries the toothpicks are tabooed. The matrons tell the girls that it is vulgar to pick their teeth. Now when the mothers tell me that, I tell them that "I don't want you to pick your teeth in the street; I don't ask your daughters to brush their teeth in the street; I don't ask them to wash their feet in the street. But I do ask them to pick their teeth after each meal. Do it carefully and in a few moments and get rid of it. Retire to the boudoir, if necessary." And have a different tooth brush than that used before breakfast, because that brush is wet. Use another one. Do that after each meal and particularly before retiring, and do not eat things between meals. Some of my patients I require to rinse their mouths two or three times between meals while being cured of pyorrhea, and if they do all that, pyorrhea can be cured and kept cured, and, as I say, they are cured when the teeth get firm.—Dr. H. Warren in *Western Journal*.

*Professional Courtesy.* One of the greatest needs in our profession today, in my opinion, is a standing together, shoulder to shoulder, for the betterment of our condition, professionally, socially and financially. We have only to take heed of the example set for us by our more humble brothers in the trades to see the tremendous advantage of co-operation (this touch of the shoulder in the ranks), over the lack of co-operation due to the small and petty jealousies and bickerings we find continually cropping out in our profession. Brothers, this uncharitableness is the greatest bane and blight of our profession today. This doctrine of hate, which some men seem to practice, is fatal to our progress. If we would bring out the best that is in us, if we would get out of our profession the best it affords for a life's devotion to it, we must think together, we must believe together, we must work together, and we must act together. It is my firm conviction that the day that sees every dentist in the State big-hearted enough and charitable enough and wise enough to see and appreciate the absolutely suicidal effect which this spirit of selfishness and self-aggrandizement has upon his chosen profession and upon him directly as a member of it, that day will be the brightest day that has ever dawned upon the profession of dentistry—Dr. H. N. Jackson in *Review.*

*Natural Remedies.* Don't rush for some remedy with which to club into insensibility every symptom of disease as soon as it puts in an appearance. Give nature a little chance to show what she intends to do before attempting to stop her by dosing yourself with some pain-reliever or colic cure. Don't trust her too blindly, for the best of things may become bad in extremes, and the body may become so panic-stricken as to keep on throwing overboard, not merely the poisons, but its necessary daily food, if the process be allowed to continue too long.

*"Stop that Cough! It is Killing you!"* Yet few things could be more obvious to even the feeblest intelligence, than that this "killing" cough is simply an attempt on the part of the body to expel and get rid of irritating materials in the upper air-passages. As long as your larynx and windpipe are inflamed or tickled by disease-germs or other poisons, your body will do its best to get rid of them by coughing, or, if they swarm on the mucous membrane of the nose, by sneezing. To attempt to stop either coughing or sneezing without removing the cause is as irrational as putting out a switch-light without closing the switch.

In the realm of the nervous system, take that commonest of all ills that afflict humanity—headache. Surely, this is not a curative symptom or a blessing in disguise, or, if so, it is ecxeedingly well disguised. And yet it unquestionably has a preventive purpose and meaning. Pain, wherever found, is nature's abrupt command, "Halt!" her imperative order to stop. When you have obeyed that command, you have taken the most important single step towards the cure. *A headache always means something*—overwork, underventilation, eye-strain, underfeeding, infection. Some error is being committed, some bad physical habit is being dropped into. There are a dozen different remedies that will stop the pain, from opium and chloroform down to the coal-tar remedies phenacetin, acetanilid, etc.) and the bromides. "But not one of them cures", in the sense of doing anything toward removing the cause. In fact, on the contrary they make the situation worse by enabling the sufferer to keep right on repeating the bad habit, deprived of nature's warning of the harm that he is doing to himself. As the penalties of this continued law-breaking pile up, he requires larger and larger doses of the deadening drug, until finally he collapses, poisoned either by his own fatigue-products or by the drugs which he has been taking to deaden him against their effect.

In fine, follow nature's hints whenever she gives them: treat pain by rest, infections by fresh air and cleanliness, the digestive disturbances by avoiding their cause and helping the food-tube to flush itself clean; keep the skin clean, the muscles hard, and the stomach well filled—and you will avoid nine-tenths of the evils which threaten the race.—Dr. Woods Hutchinson.

*The Technique of Investing as Given by Dr. Taggart.* In an ordinary plaster bowl place a certain quantity of water (it should be pure water), the quantity depending upon the amount of investing material needed to fill the flask you are using; into this water sift the requisite amount of investment compound, and with a spatula stir to thoroughly mix. Then, inclining the plaster bowl to one side, tap it on a bench or table and slowly rotate. This will cause the material to spread itself in a thin coating over most of the inner surface of the plaster bowl and will cause an elimimation of most of the air contained in the mixture. Continue this tapping and rotating until you are satisfied most of the air is out.

The wax model having been previously mounted on a sprue and crucible former, is now carefully coated with the investment material. Now thoroughly cover the model, sprue and crucible former, then carefully place the flask and fill to excess with investment.

Holding the crucible former or lid securely on the flask, invert it into a glass slab and slowly and carefully press it down so that the investment material will thoroughly pack around the model, slide the flask off the glass slab, now over a little more investment material on the slab, press it down again and slide off again; repeat this several tines to make sure it is well packed.

A small hole through one side of the flask about 2mm. in diameter is of advantage in that, as the flask is pressed onto the slab, the excess comes out of this hole and any air that may have been caught while investing will often be gotten rid of through this hole, as the excess of material runs out through it. The last time pressure is brought on the investment place your finger over the hole; this will make it pack more solidly.

Care must, of course, be exercised, and the work must be rapidly done, so that the investment material does not get too stiff. Now, carefully dry and burn out the wax. Do not overheat the flask, after it is thoroughly dry, let the flask cool and then make the casting.—Dr. G. W. Dittmar in *Review*.

*A Cast Backing for a Porcelain Faced Crown.* With root prepared a band is made in the usual manner and facing ground and fitted as for Richmond crown. The wire to be used for dowel is hammered out flat on one end and notched to correspond with facing pins so that the flattened end will pass in between them and lay flat against the facing. The other end of dowel is adjusted to canal so that facing with pins resting in notches of dowel will fit snugly against band labially and line up with other teeth. The facing is now removed from the mouth and a mass of softened wax is forced around dowel base and back of facing and with base end of wax again softened over flame, force to place on root. The band which was left on root should now be removed with dowel facing and wax all intact and secured to place by running a hot instrument around the outer edge, melting the wax to it. Remove excess wax, warm up slightly and put back onto root for final adjustment, after which carefully remove from the mouth and with a piece of sticky wax heated and attached to facing pull it away from wax, place carbon points in pin holes, invest and cast. The facing may be secured to the backing by one of two means. If backing is thick enough to cover the full length of pin the facing can be securely attached in the following manner: With a twist drill two or three sizes larger than pin holes, drill out carbon points, thus making holes slightly larger than pins to accommodate a sort of head that is made on end of pins by pinching with pliers. The holes are now carefully filled with cement and with entire back of facing covered with cement it is pressed to place.

In case of thin backing the holes should extend through to the lingual surface and slightly countersunk. Pins are cut off flush with surface of backing and facing is secured to backing by cementation and riveting, which should be done before the cement crystallizes by imbedding facing in a softened mass of modeling compound with backing up, and with mall jeweler's hammer rivet ends of pins and finish flush with lingual surface of backing. This method offers an absolutely reliable means of attaching facing to thinnest of backing, is easily accomplished, and in case of breakage is repaired with equal facility.—F. E. Roach in Nov. 1910 *Review.*

*How Mr. Fletcher Eats.* There is something about Mr. Fletcher's method of mastication that has never been properly described, so far as the editor has seen, and he has read much about it. The statement is always made that mastication should be continued till the food is almost liquid and will practically swallow itself, and emphasis is laid on chewing it till the taste is extracted from it. This would naturally imply long-continued mastication, but in the intelligent application of the process this is not necessary. It would be better to state that on taking the food into the mouth the chief effort is concentrated on searching out every minute particle with the tongue and tasting it. In doing this it will be found that the tongue is very active and it really becomes a most important factor in the process of mastication. In the deliberate attempt to taste, the tongue forces its way through the food and crushes it against the lingual surfaces of all the teeth, upper and lower, and thus a large mass of food is being comminuted at once. Then, again, this concentration on the taste stimulates the salivary glands and a flood of saliva is poured into the mouth, which rapidly liquifies the food and it is swallowed in spite of the individual. This is an explanation of Mr. Fletcher's statement that the food should be chewed till it swallows itself. It will be found impossible not to swallow frequently when the food is prepared in this way.

The deliberate tasting of every particle of the food prevents the swallowing of any portion which may be tainted or in any way unwholesome, and thus nothing goes into the stomach which is deleterious. There is much of the meat furnished consumers which will not stand this test, and this accounts for the fact that Mr. Fletcher and his disciples largely eschew a meat diet.—Dr. C. N. Johnson in Dec. 1910 *Review*.

*Incisal Cavity Preparation.* There are altogether too many men in the dental profession who sacrifice the stability of their operations for esthetic reasons. The idea of making dental or other operations is to make them permanent. In operating on the proximal surfaces of the upper incisors and cuspids there are many operators who do not extend the cavity margins sufficiently incisally to involve the point of contact. The result is that, later on, we have a cavity of decay at that particular part of the tooth. Had ample separation been obtained on the start, there would have been plenty of convenience, so that the operation could have been made in a proper manner and recurrence of decay obviated.

I have been humiliated by the failures that followed my operations which were made in the proximo-incisal surfaces. My method of cavity prepararion in these cases usually is the step cavity, and I feel that it is the most satisfactory preparation for this class of operations. In the preparation of these cavities my former method was to bring the incisal and proximal walls to rather sharp angles. This form of preparation was fairly successful in those teeth that carry their size linguo-labial well to the incisal surface, especially where they present a broad surface from wear; but in those cases where the tooth is long and thin failure very frequently marks the result of our labor. But failure is no discredit to any man, but the failure to profit by these mistakes is indeed a discredit to all; hence, let us look at our failures, study the conditions presented by them and try to avoid stumbling over the same obstacle when we come in contact with it again. In the particular class of cavities referred to we find the failure occurs at the angle of the incisal and proximal surfaces, and upon a closer examination we find the enamel rods extending well out to the angle are supported by little, if any, dentin, so that in condensing gold against them we are likely to cause fracture, and later find them falling out or moisture penetrating them, leaving the usual dark line. The angle upon the lingual surface is also a source of danger from the fact that the constant impact of the lower teeth against the lingual surface of the upper teeth is a menace to enamel rods which are not well supported by dentin. In order that the weak points of the operation may be removed, I cut the enamel both lingually and labially at this point and round the angle of the cavity instead of leaving it square—Dr. Wallace in *Brief*.

*The Gold Inlay.* It is never necessary to remove any part of the tooth structure for an inlay that ought not to be removed to secure proper restoration by any method whatever. The fact is that the inlay has done more to impress and emphazise correct principles of cavity formation than anything else ever said or done. A cavity prepared for an inlay is practically the same in every instance that it should be for dependable restoration either with gold foil or amalgam.

The answer of my objector will be that the preparation for the inlay requires that the wax impression must "draw," or more properly, be drawn, so as to preserve its form intact, whereas in preparation for foil or amalgam undercuts may be left, and overhanging tooth substance preserved. I will answer that in any case where an undercut is permissible or an over jutting wall *should* be left the undercut may be filled with cement and the walls paralelled for the inlay impression.

If anyone will study darefully the cuts and diagrams of cavity preparation as put forth by our first authority in such matters, Dr. Black, he will discover that in every instance the formation for metallic fillings is almost ideal for inlays in corresponding situations. There is the same flat base, parallel walls, beveled margins, the removal of all projections of doubtful strength, "extension for prevention." In this connection the chief error consists *not* in vandalism for the *inlay,* but in faulty preparation nine times out of ten for the filling. Walls are left that should be removed; cusps weakly supported are sentimentally allowed to remain that should be cut off, down, down to a solid base, to "bedrock," for the due support of a metal substitute.

I employ the "Ivory" matrix to secure form and resistance, for we must in every case be able to press the wax into every groove, and over every margin. Cool, remove matrix, go over all margins with trimmers and burnishers just slightly warmed. Touch the occlusal surface with a bit of warm air and have the patient close the teeth. Be sure the occluding teeth are wet; trim, burnish, chill, remove with a deeply inserted fine point (a stiff explorer is good), reinsert, go over margins again, remove, attach sprue and proceed to invest.

As a last step, however, previous to investment I go over the cavity side of the wax impression with sharp spoon excavators, or burs with the engines, so that the cement shall have here and there a better grip, and to make sure that the fit shall not be so close as to prevent the *edges* of the inlay coming absolutely to contact with the enamel margins. As to durability, we do not have fifty years of record to fall back on for evidence. As to cast inlay, every process must have its beginning. But we do have the evidence of fifteen or twenty years for the inlay, as made with the burnishing method by Dr. Ames, of Chicago, and others. And I know that their work has "stood up." I have been putting in cast inlays for several years, at first with very imperfect appliances. There must have been a good many scores of them, and so far as I know, not one has failed yet. Any one may be successful if he will but remember that there are no unimportant details. Here as elsewhere, "If you do not take pains, pains will take you."

And let me say in conclusion, the cast gold inlay is not the enemy or antagonist of the foil filling; it is rather the adversary of amalgam; also of the "shell" crown, in places where real vandalism is so often perpetrated to give it room. —Dr. Garrett Newkirk in *Review.*

